# Normal Adult Height in an Untreated Boy With McCune-Albright Syndrome Presenting With Precocious Puberty

**DOI:** 10.1016/j.aace.2023.02.007

**Published:** 2023-03-05

**Authors:** Ugen Lhamu, Sabitha Sasidharan Pillai, Anna Delamerced, Jose Bernardo Quintos

**Affiliations:** 1 Division of Pediatric Endocrinology and Diabetes, Department of Pediatrics, The Warren Alpert Medical School of Brown University/Rhode Island Hospital/Hasbro Children’s Hospital, Providence, Rhode Island; 2 Department of Pediatrics, Yale School of Medicine, New Haven, Connecticut; 3 Division of Pediatric Endocrinology and Diabetes, Department of Pediatrics, The Warren Alpert Medical School of Brown University, Providence, Rhode Island

**Keywords:** boy, McCune-Albright syndrome, precocious puberty, height

## Abstract

**Background/Objective:**

We present a boy with McCune-Albright syndrome (MAS)-associated precocious puberty (PP) who achieved normal adult height without treatment.

**Case Report:**

The patient presented at 10 years of age with PP and fibrous dysplasia of the right humerus. Examination showed a height 148.7 cm, Tanner 2 pubic hair and 12-15 cc testes. The Bone age (BA) was 13 years with a predicted adult height of 175 cm vs. mid parental target height of 173 cm. Laboratory parameters were as follows: luteinizing hormone (LH) 0.745 mIU/mL (0.2-4.9 mIU/mL), follicle stimulating hormone (FSH) 0.933 mIU/mL (1.8-3.2 mIU/mL), testosterone 42 ng/dL (18-150 ng/dL), inhibin B 436.6 pg/mL (41-238 pg/mL) and AMH 36.1 ng/mL (45.26-191.34 ng/mL). The DNA testing result of tissue from the right humerus was positive for *GNAS* p. R201C mutation confirming a diagnosis of MAS. Pubertal progression with growth spurt occurred over the next 3 years: growth velocity (GV) 12 cm/y, testosterone 116 ng/dL, LH 0.715 mIU/mL and FSH 1.3 mIU/mL at 10.6 years; GV 10.3 cm/y, BA 13 to 13.6 years, testosterone 450 ng/dL, LH 1.7 mIU/mL and FSH 1.4 mIU/mL at 11.7 years; and GV 3.8 cm/y, BA 17 years, Testosterone 668 ng/dL and LH 4.2 μIU/mL at 13.3 years. Height was 171.2 cm.

**Discussion:**

PP is reported in approximately 15% of boys with MAS. PP leads to BA advancement and reduction in final adult height. Our patient achieved normal adult height without treatment in the absence of excess growth hormone.

**Conclusion:**

Boys with MAS and PP with slow BA advancement may achieve normal adult height without treatment even in the absence of excess growth hormone.

**Highlights**

• A male with McCune-Albright syndrome (MAS) and precocious puberty (PP)
• Growth, puberty, and bone age should be monitored closely in boys with MAS and PP
• Treatment may not be needed to achieve normal height in some boys with MAS and PP

**Clinical Relevance**

Close monitoring of growth, puberty, and bone age in boys with McCune-Albright syndrome (MAS) and precocious puberty (PP) is needed. Treatment may not always be needed to achieve normal adult height in some patients. Additional cases are needed to increase knowledge of management of MAS-associated PP in males, and longitudinal investigations are warranted to track patients to final height.

## Introduction

McCune-Albright syndrome (MAS) is a rare disorder due to a postzygotic somatic mutation in the *GNAS1* gene characterized by a triad of fibrous dysplasia, café-au-lait spots, and peripheral precocious puberty (PPP).^1^ Patients with MAS may develop multiple endocrinopathies, such as hyperthyroidism, pituitary adenomas, and excess growth hormone (GH).^1^ PPP is rare in males compared with females and has been reported in approximately 15% of boys with MAS.^2^ Precocious puberty (PP) leads to bone age (BA) advancement, premature epiphyseal closure, and reduction in final adult height.^3^ A child’s adult height potential is estimated by calculating the midparental height (MPH), which is a child’s projected adult height based on the heights of the parents. MPH for boys is calculated by adding the average of parents’ heights with 6.5 cm and is considered normal for the family if the child’s estimated final height is within 5 cm of the MPH.^4^

Limited data are available on the management of boys with MAS and PPP. All prior reports show achievement of adult height with treatment including aromatase inhibitors or antiandrogens that block androgen synthesis or action with addition of gonadotropin-releasing hormone (GnRH) analogs with the development of central PP.^2^^,^^5^, ^6^, ^7^, ^8^ Patients who were not treated had a very short stature if there was no excess GH.^5^ We report a case of a 10-year-old boy with MAS who achieved adult height within his MPH without treatment in the absence of excess GH.

## Case Report

A 10-year-old boy was seen in the Pediatric Endocrinology Clinic for evaluation of PP. He had adult-type body odor at 9.1 years and pubic hair at 10 years. He was diagnosed with fibrous dysplasia of the right humerus at 9.11 years when evaluated for right arm pain at the emergency department. X-ray and magnetic resonance imaging with contrast of the right humerus showed a 6.7-cm × 2.2-cm × 2-cm expansile lesion in the central portion of the diaphysis of the humerus. Leading differentials at this point were fibrous dysplasia, eosinophilic granuloma, and chronic osteomyelitis. Biopsy of the lesion showed fibrous dysplasia. He also had a history of fracture of the left forearm at the age of 5 years after a fall from a slide while playing, with x-ray showing left supracondylar and distal ulnar fractures. There was no family history of PP. His height was 148.7 cm (SD score [SDS], 1.45), and he had no café-au-lait spots. The thyroid gland was not enlarged. He had Tanner 2 pubic hair, a left testicle of 15 mL, a right testicle of 12 mL, and Tanner 2 genitalia with a stretched penile length of 5 cm. The BA was 13 years, with a predicted adult height of 175 cm versus midparental target height of 173 cm. Laboratory parameters were as follows: luteinizing hormone (LH), 0.745 mIU/mL (0.2-4.9 mIU/mL); follicle-stimulating hormone, 0.933 mIU/mL (1.8-3.2 mIU/mL); testosterone, 42 ng/dL (18-150 ng/dL); inhibin B, 436.6 pg/mL (41-238 pg/mL); anti-Müllerian hormone, 36.1 ng/mL (45.26-191.34 ng/mL; mean, 92.52 ng/mL); and β human chorionic gonadotropin, <2 mIU/mL. His insulin-like growth factor 1 level was 280 ng/mL (106-443 ng/mL) and thyroid stimulating hormone level was 2.33 μIU/mL (0.35-5.5 μIU/mL). Increased uptake was observed in the midshaft of the right humerus, distal right radius, and superolateral right scapula on a nuclear medicine whole body scan. Testicular ultrasound revealed no abnormality. The DNA testing result of tissue from the right humerus was positive for *GNAS* p. R201C mutation, confirming a diagnosis of MAS.

He was seen in our clinic every 6 months. The Table summarizes the 3-year endocrine follow-up. At his last visit at 17 years, his height was 172.9 cm and both testes were >30 mL with Tanner 5 pubic hair and genitalia. His laboratory parameters were LH of 5.1 mIU/mL, follicle-stimulating hormone of 1 mIU/mL, testosterone of 564 ng/dL, insulin-like growth factor 1 of 212 ng/mL, and thyroid stimulating hormone of 0.91μIU/mL.

**Table:** Auxological, Clinical, and Biochemical Data of a Boy With McCune-Albright Syndrome and Sexual Precocity

	May 8, 2015	December 2, 2015	June 8, 2016	December 7, 2016	June 7, 2017	January 25, 2018	July 26, 2018	October 30, 2019
CA (y)	10	10.7	11	11.7	12	12.9	13.3	14.6
BA (y)	13	13	…	13-13.6	…	…	17	…
Height (cm)	148.7	155.2	160.5	165.6	167.3	169.3	171.1	171.9
Height (SDS)	1.45	1.96	2.22	2.47	2.23	1.88	1.62	0.56
GV (cm/y)	…	12	9.67	10.3	3.4	3.2	3.8	0.5
MPH (cm)	173	173	173	173	173	173	173	173
APH (cm)	174	182	…	182	…	…	171	…
Right testis (mL)	12	20	20-25	20-25	20-25	…	25	25
Left testis (mL)	15	20	20-25	20-25	20-25	…	25	25-30
Pubic hair	Tanner 2	Tanner 3	Tanner 3	Tanner 4	Tanner 5	…	…	Tanner 5
Testosterone (ng/dL)	42 (18-150)	116 (200-620)	…	450 (350-970)	420 (350-970)	…	668 (350-970)	…
FSH (mIU/mL)	0.933 (1.8-3.2)	1.3 (2.6-11)	…	1.4 (2.6-11)	1.6 (2.6-11)	…	1.3 (2.6-11)	…
LH (mIU/mL)	0.754 (0.2-4.9)	0.715 (0.4-7)	…	1.7 (0.4-7)	…	…	4.2 (0.4-7)	…
Inhibin B (pg/mL)	436.6 (50-310)	256 (41-328)	…	…	…	…	…	…
AMH (ng/mL)	36.1 (45.26-191.34)	…	…	…	…	…	…	…
β-hCG (mIU/mL)	<2	…	…	…	…	…	…	…
IGF-1 (ng/mL)	…	…	…	330 (86-393)	280 (265-652)	…	263 (265-652)	200 (220-574)

Abbreviations: AMH = anti-Müllerian hormone; APH = adult predicted height; BA = bone age; CA = chronological age; FSH = follicle-stimulating hormone; GV = growth velocity; hCG = human chorionic gonadotropin; IGF-1 = insulin-like growth factor 1; LH = luteinizing hormone; MPH = midparental height; SDS = SD score.

## Discussion

We report a 10-year-old boy with MAS who presented with bilateral macroorchidism, fibrous dysplasia, absence of café-au-lait-spots, and positive *GNAS* mutation. He later developed PP with rise in serum testosterone to adult levels and subsequent BA advancement. He achieved adult height consistent with his midparental target height without treatment.

The phenotype of MAS is mostly decided by *GNAS* mutation expressing cells and the pathophysiologic effects of mutation on these tissues. Approximately 30% of patients show the classic triad, whereas 70% show 1 or 2 of the cardinal features that include café-au-lait spots, PPP, excess GH, excess prolactin, hyperthyroidism, or hypercortisolism in addition to polyostotic fibrous dysplasia.^1^^,^^2^

Our patient presented at 10 years of age with Tanner 3 to 4 testicular volume (TV) (a 12 -mL right testicle and a 15-mL left testicle) discordant with a Tanner 2 penis and pubic hair. His LH (0.75 mIU/mL) and testosterone (42 ng/dL) levels were also consistent with Tanner 2 puberty for males. TV is mostly a reflection of seminiferous tubules, and increased testosterone secretion from Leydig cells at puberty is responsible for the development of secondary sex characters. Expression of *GNAS* mutation in the Sertoli cells can cause macroorchidism due to Sertoli cell proliferation and hyperfunction with increased concentration of serum inhibin B and anti-Müllerian hormone.^9^ Although we did not perform genetic testing for *GNAS* mutation in the gonads, our patient’s macroorchidism without corresponding penile enlargement is more likely due to Sertoli cell activation. He also had elevated inhibin B levels (436 pg/mL) suggestive of Sertoli cell activation. Macroorchidism was reported in 13 of 26 children with MAS in a study.^5^

During the second visit 7 months later, our patient had a Tanner 5 TV and testosterone level of 116 ng/dL without an increase in LH level (0.715 mIU/mL), which might indicate gonadotropin-independent PP secondary to autonomous hyperfunction of Leydig cells. Disruption to Leydig cell development due to *GNAS* mutation in MAS may lead to autonomous Leydig cell maturation and increased testosterone production in childhood, resulting in PP.^5^ Given BA, which had remained unchanged since 13 years at initial presentation, a decision to closely follow his puberty and BA was made. Follow-up laboratory evaluation 1 year later (11 years and 7 months of age) showed a rapid rise in serum testosterone from 116 to 450 ng/dL and LH level from 0.715 to 1.7 μIU/mL, suggesting hypothalamic–pituitary–gonadal (HPG) axis activation. Gonadotropin-independent puberty might have led to secondary central PP in our patient. His BA had advanced slightly to 13.6 years during this interval. Messina et al^2^ reported a boy with MAS who presented with unilateral macroorchidism due to Sertoli cell activation with subsequent activation of Leydig cells and HPG axis, similar to our patient.

Given the rarity of PP in males with MAS, limited data are available on the management of these boys with PP. Therapeutic modalities that have been used in boys with MAS and PP include a combination of antiandrogens to block the androgen action at the receptor level (bicalutamide, flutamide, cyproterone acetate, and spironolactone) and aromatase inhibitors (anastrozole and testolactone), which blocks the conversion of androgens to estrogens or antiandrogen, which in turn blocks adrenal and testicular androgen biosynthesis (ketoconazole). GnRH analogs are added in cases with secondary HPG axis activation.^2^^,^^5^, ^6^, ^7^, ^8^ A study on 54 males with MAS, including 26 boys, observed PP in 11 subjects who presented between ages 2 and 8 years.^5^ Two patients entered puberty by 9 years and reached final height at a young age. Both these patients had premature epiphyseal closure. One had a very short stature and the other one with excess uncontrolled biochemical GH reached a height SDS equal to MPH. Five patients who were treated with a combination of aromatase inhibitors and androgen receptor blockers with addition of leuprolide at the onset of central PP showed improvement in predicted adult height. Two adults with excess GH reached a final height above 0 SDS without treatment, whereas 1 untreated adult without excess GH had a very short stature.^5^ Ten boys without PP entered puberty at a median age of 11 years in this study.^5^ However, no data on the final height of patients without PP were available. Other single case reports described successful management of boys with MAS and PP with antiandrogens with or without GnRH agonists.^2^^,^^6^^,^^7^

In contrast to prior reports, our patient achieved an adult height within his genetic potential without treatment.^2^^,^^5^, ^6^, ^7^, ^8^ He had no other endocrinopathies, including excess GH. The decision to monitor the growth and puberty closely was taken for our patient given the slow progression of BA despite robust growth velocity, and he received no treatment to suppress the puberty.

To the best of our knowledge, this is the first report of a young male patient with MAS and PP who achieved normal adult height without treatment in the absence of excess concomitant GH. Close monitoring is needed to follow up on growth, pubertal progression, and BA advancement. Treatment may not always be needed to achieve normal adult height in patients with slow advancement of BA, as in our patient. Additional cases are needed to increase the knowledge on management of MAS-associated PP in males, and longitudinal investigations are warranted to track patients to final height.

## Disclosure

The authors have no multiplicity of interest to disclose.
